# Causes and Cure of Disease

**Published:** 1886-07

**Authors:** 


### "CAUSE AND CURE OF DISEASE"

The above is the title of a work just published by H. B. Philbrook, the author and publisher, who is also editor of a serial entitled *Problems of Nature,* which has fairly taken the votaries of popular science by the ears.

The author in his preface, is neither timid nor hesitating, but comes directly to the front with a clear ringing challenge to physicians of the old school, who rely upon mineral poisons in the treatment of disease. He admits that the schooled practitioner may be useful in pointing out the specific ailment of the afflicted, but when he has gone so far he refers to the work in question as the better doctor of the two, and proceeds to inform us that no physician has been consulted in its preparation and no medical work except to obtain the ample list of diseases which precedes the text, of every one of which the *cause* is given, the disease scientifically diagnosed and a remedy prescribed not in accordance with any recognized school of medicine, as a natural and necessary sequence to his premises, for if his theory of the manifold diseases of which he treats is correct ; his methods of cure follow as simply and naturally as the use of water to supply moisture or of heat to carry off an excess of it. If we correctly understand him, he traces the origin of every disease which "flesh is heir to" either to a deficiency or over supply of life elements, and repudiates as an old superstition, the theory that latent poisons lurk in a healthy system ready to actively manifest themselves upon the slightest encouragement. To use his own words, "this old superstition that a latent poison is in the system of all persons is as wise as the claim of a church that a devil is latent in all nature and only wants a chance to show himself."

In allusion to the mind cure craze, he says : "A clever woman in a pulpit of a superstitious church is giving people a still more astonishing disclosure of a process of cure, and it is exactly the same affair or character of cure, as a priest of every country of the old world was offering when a church monopolized the consideration of diseases through the power of a priest." An acquaintance with the author of a work, whether literary or scientific, is always a source of additional interest to the student or reader. In the present instance we are able to assure our readers that whether right or wrong, the author of the work under review is in sober earnest and means all he says. Having at an early period of his life adopted what prior to the adoption of the civil code, and an elective judiciary was classed as one of the three so called learned professions, he found time to investigate the claims of scientists to a knowledge of natural things, only to differ so widely from nearly all accepted theories, as to entitle his opposing views to the claim of originality, at the very least. The immediate outcome was the establishing, editing, and publishing, single handed of a weekly journal entitled "Problems of Nature," in which the author (for there was really but one) made bold to attack almost everything in popular science, within telescopic or microscopic range, and propound an entirely new system of philosophy in accordance with what we might properly term his *interior* perception. The work in hand is characterized by great clearness of statement, and unwavering confidence in the soundness of views presented in such direct divergence from the well worn ruts of other systems, that if either is right the other must be wrong.

The book is published at the office of "Problems of Nature," and sold at the moderate price of Two dollars. As a sanitary guide merely, it is worth more than its price.

### URETHANE.

Some months since, the attention of the profession was directed to a new hypnotic and sedative called Urethane. It was greatly extoled, and claims made for it which seem to be fully sustained by repeated experiments and use.

The conclusions reached are that we have a powerful and yet very safe hypnotic. That it is very agreeable to take and does not interfere with any of the functions of the body. That upon its administration, calm sleep, free from dreams, follows in about ten to forty minutes. Some observers claim to have seen patients under the influence of the drug from twenty-four to forty-eight hours after one administration. All agree that it is not rapidly eliminated from the system. This is a fact we must bear in mind in its use, although it can be tolerated in large doses. It seems to be an established fact that the dose of this drug is from twenty to sixty grains, once or twice daily. "It is clear that the principal action of urethane is on the brain, without producing any marked irritation of the peripheral or sensory apparatus, consequently it is useless in the treatment of neuralgic pains, as well as in the pains of locomotor ataxia. But in other conditions where sleeplessness is the main symptom to be combated, urethane seems to possess the greatest advantages, since it is well borne by the patient; it produces absolutely no unpleasant symptoms, and the sleep which it provides is identical with normal physiological sleep. It would also appear that this remedy is particularly suitable for use in the treatment of diseases of children, where the need of a safe and sure hypnotic is greatly felt." We see from various reports that all agree it is well adapted to the treatment of insomnia, associated with heart and pulmonary troubles. It has a peculiar effect in insomnia, associated with this former class of diseases. There is a difference of opinion as to its action on the human system. Some observers claim that it acts simply on the cerebrum, and that it does not affect the respiratory or circulatory apparatus; while others say it acts on the spinal cord, and by its use the number of heart-beats and respirations are reduced. This seems to be conclusively proven, although the former view is sustained by many prominent men, after careful research and investigation with the drug. Prof. Coze has shown that, physiologically, it is an antidote for strychnine.

At a recent meeting of the Medical Society in this city, an interesting paper was read on Urethane, the author reported many cases of sleeplessness treated by its use, with invariable good results. Those present related the successful use of the remedy in many cases of insomnia, associated with various diseases. The only failure reported was in a case of hysteria, where all remedies failed. In addition to the treatment of insomnia, we find it applied by Prof. Coze to the treatment of convulsions, especially tetanus. We should then give this new remedy a trial in epilepsy, chorea, tetanus and migraine; if it be found to be as valuable in this field as in that in which it already has a place, it will be a great boon to the profession.—*Buffalo Med. Surg. Journal.*

